# Outcome Determining Factors for displaced Intra-articular Calcaneal Fractures treated operatively

**DOI:** 10.5704/MOJ.1511.001

**Published:** 2015-11

**Authors:** SA Nawfar, KL Chan, HM Idham, IM Izani, T Nahulan

**Affiliations:** Department of Orthopaedics, Universiti Sains Malaysia, Kubang Kerian, Malaysia; * Department of Orthopaedics, Queen Elizabeth Hospital, Kota Kinabalu, Malaysia

**Keywords:** Intra-articular calcaneal fracture, Maryland Foot Score, SF-36v2 health survey

## Abstract

Introduction: Calcaneal fractures are caused by high energy trauma and mostly are intra-articular fractures. Nondisplaced intra-articular calcaneal fracture (IACF) can be treated non-operatively. However, displaced intra-articular need to be reduced and fixed anatomically to facilitate early ankle rehabilitation and minimize functional impairment. This study was done to find out the outcome of the IACF patients who underwent operative treatment.

Methods: 62 patients with IACF were selected in this study and had been followed up from June 2009 to May 2013. They were placed into two groups; the operative treated and non-operative treated groups. Bilateral ankle lateral view plain radiographs were taken for comparison of the Bohler and Gissane angles. Both groups of patients were assessed by the Maryland Foot Score (MFS) and the SF-36v2 general health survey questionnaire. The ability of the patients to perform activity of daily living (ADL) and /or return to work (RTW) was assessed as well.

Results: The operative treatment group of displaced IACF patients achieved no significant better scores in the mean MFS and SF-36v2 mean scores as compared to non operated cases. There was no difference in RTW between the 2 groups, but earlier ADL was recorded in the operated group. However, this study had found 5 associated factors which causes major effect to the patients’ outcome to treatment.

Conclusions: The patient’s compliance with post-operative rehabilitation regimen were found to be significantly related with the outcomes.

## Introduction

Calcaneal fractures account for 60% of all tarsal bone fractures^1-3^. They are caused by high energy trauma in which it usually occurs by axial load on the patient’s heel^4, 5^. 75% of calcaneal fractures are intra-articular fractures. Men commonly sustain this type of injuries compared to women because it commonly occurs as occupation associated injuries^6^. IACF can be treated either with or without surgery depending on severity.

The aim of this study was to assess and to compare the outcome of both non-displaced and displaced IACF that were treated surgically, for a period of at least 6 months post-trauma by using objective and subjective assessment measurement tools. It was for the patients treated at Queen Elizabeth Hospital in Kota Kinabalu, Sabah. The Maryland Foot Score (MFS) was used as an objective and the General Health (Short Form-36 [SF-36v2]) was used as a subjective assessment tool for patient’s general health status. The ability of surgically treated groups were compared with the non displaced fractures which were treated non operatively for the ability to return back to work (RTW) and perform activities of daily living (ADLs). Factors which affect the outcomes of IACF patients was hoped to be elucidated.

Displaced IACF can lead to bad consequences if the fracture displacement was not reduced and fixed anatomically. Many authors suggest that an anatomical reduction of the displaced IACF cannot be accomplished using non-operative methods and instead recommend surgery^7-9^. The selection of operative intervention usually was influenced by the severity of fractures, fracture patterns, the soft tissue conditions, and also the patient’s underlying pre-morbid illnesses^10^. There are generally more than 15 types and combinations of treatment methods for IACF^11-14^. Open reduction and internal fixation (ORIF) is the most commonly used technique for calcaneal fracture fixation, mainly via the extended lateral approach^15-17^; and is the best method of achieving anatomic joint reduction and calcaneus morphology restoration. In addition, the soft tissue complications are proportionally direct with the aggression magnitude of soft tissue^18^. Intra-operative C-arm fluoroscopes imaging were also carried out to assess precisely the subtalar joint congruity^19^. Osteosynthesis implant selection and fixation was up to the surgeon’s discretion and the fracture characteristic^20-23^. The usage of bone grafts or bone substitutes are still controversial^18,24^. Most authors suggested post-operative non-weight bearing for 6 weeks^16,17,25,26^; after which the patient was allowed partial weight bearing.

## Materials and Methods

The cases were retrospectively identified and collected from those patients who had sustained IACF from Jun 2009 to May 2013 and were treated in Queen Elizabeth Hospital, Kota Kinabalu, Sabah. The calculated total sample size required was 51 patients. Patients who had sustained unilateral closed IACF classified using Sander’s Type I, II, III or IV injury without concomitant injuries in the ipsilateral limb were selected. The patients were advised not to bear weight over their fractured foot until radiological evidence of union was seen. All these patients had at least 6 months treatment before assessed by the investigator. Patients’ demographic data were recorded and bilateral ankle lateral view plain radiographs were taken to measure the Bohler and Gissane angles. Only radiologically displaced fractures were subjected to CT scan. The patients were invited to complete the SF-36v2 health survey form; and their symptoms and disability were assessed by the MFS.

Patients having bilateral calcaneal fractures; open fractures; soft tissue compromise or crush heel pad which prevented timely surgery; compartment syndrome; wound or fracture healing complicated by infection and osteomyelitis; peripheral vascular disease or neurological injuries to either lower limb such as sciatic nerve injury, paraplegia or hemiplegia; and psychiatric illnesses, non-ambulatory or demented patients were excluded from the study. Patients who having symptoms of Regional Sympathetic Dystrophy (RSD), peroneal tendinitis, impingement or entrapment; having multiple surgeries at the same site such as revision surgery, bone grafting for delayed union or removal of implants; taking prolonged medications such as corticosteroids, anabolic steroids, diuretics, cytotoxic drugs and anticonvulsive; and those patients who had comorbidities and metabolism disorders such as ESRF (end-stage renal failure) on haemodialysis, rheumatoid arthritis, liver disease, poorly-controlled diabetes, malignancies, endocrine abnormalities (parathyroid/thyroid glands, adrenals), total/partial gastrectomy or post-ovariectomy status; were also excluded from this study.

Non-operative treatment involves rest, cooling pack, compressive bandage and elevation 25. A simple posterior, removable, well-padded splint applied in a plantigrade position to avoid equinus contracture and potential skin complications was used. Non-steroidal anti-inflammatories were used for pain relief. The injuries will then become comfortable within a week or two for early physiotherapy and range of motion exercises for of all joints to prevent stiffness^26,27^. Non-weight bearing was maintained until evidence of healing, which occurs after some 8 to 12 weeks 1, 28. Beyond that, weight bearing was progressively applied^29^.

The operative method used in the patients of this study was using the calcaneal locking compression plate by DepuySynthes^®^.

Sander *et al.*^30^ mentioned that the radiological assessment should assess for return of body height, width, and length by comparison with the normal side, anatomical reduction of articular surface of the posterior facet, and restoration of Bohler and Gissane angle. The lateral view is useful for measuring Bohler and Gissane angle; assessing loss of calcaneal inclination and evaluating involvement of the subtalar joint^31^. The Bohler angle reflects the relationship of the posterior facet relative to the tuberosity of the calcaneum and it effectively describes the energy of the injury^31^. The Gissane angle is seen directly inferior to the lateral process of the talus and is represented by two strong cortical struts that extend laterally. It is more specific for evaluate the subtalar joint intra-articular distortion and predict gait difficulty. The non-fractured calcaneal parameters were used as a control for radiological assessment. There were resulted in three groups of patients: “Anatomical” was defined as those anatomical reduction and fixation of fractures with no articular incongruity or whatsoever; “Near-anatomical” were those reduction and fixation of fractures with less than 3mm of articular incongruity or gapping between fragments and more than 5° but less than 10° different from the same patient’s contra-lateral non-fractured foot normal Bohler and Gissane angles; “Approximate/failure” were those reduction and fixation of fractures with more than 3mm of articular incongruity or gapping between fragments and more than 10° different from the same patient’s contra-lateral non-fractured foot normal Bohler and Gissane angles^26,30^.

Sanders classification has become widely accepted in the evaluation of intra-articular fractures based on CT scans^32^. It has ease of description for the fracture patterns and with the CT reconstruction; it gives precisely the location and number of fracture lines through the posterior facet for planning the operative intervention^30^. It also correlates better with the prognosis and outcomes^32,33^. However, the routine use of post-operative CT scan evaluation is not recommended in clinical practice or for research purposes^34^ and may not be a practicable method in view of artefacts of the bone-metal interface, unnecessary x-ray exposure, expensive and creating a dilemma between doctor & patients in case of fair or failure of anatomical fixation and medical legality. Only patients with displaced displaced fractures requiring surgery were subjected to CT scan in the current study.

In this present study, all patients were assessed by using MFS and SF-36v2 general health status questionnaires. The MFS has broad current acceptance^35^ and it has content validity for pain and physical function. In addition, the MFS showed better correlation with the concomitant use of SF-36 in the assessment of patient’s outcome when compared to the AOFAS Ankle Hindfoot Scale^35,36^. In MFS, it has 3 components which are the pain (45 marks), the functions (55 marks) and the motion (5 marks); and total maximum of 100 points. The patient is considered to be excellent if scores 90-100 points; good if scores 75-89 points; fair if scores 50-74 points; and considered failure if less than 50 points.

SF-36v2 is a multipurpose, short form health survey with only 36 questions. It yields an 8-scale profile of functional health and well-being scores as well as psychometrically-based physical and mental health summary measures^36^. The Malay version of SF-36v2 has its generally acceptable internal consistency and validity^37^ and is widely accepted. The SF-36v2 has eight scales that gauge eight domains of HRQoL, which represented by the Physical Component Summary (PCS) and Mental Component Summary (MCS). Total mean scores for each domain ranged from 0 to 100, and the higher mean scores suggested the better HRQoL^38^. If a scale score is below 50, the health status is below average. These scores are easier to interpret and simpler to analyse statistically^39,40^.

## Results

There were 62 selected patients with IACF from Jun 2009 to May 2013. The age of the cases ranged between 14 to 74 years old, with median of 40 years old (Table I). All patients had been followed up ranged from 6 to 48 months (median of 18.5 months) after receiving definitive treatment (post-treatment duration assessment). Cases were predominantly male (83.9%) and of Malay ethnicity (41.9%). Non-heavy labourer worker comprised 87.1% of patients where 64.5% of the patients fell down from heights. The right foot was the most injured foot (72.6%) compared to the left foot. Most of the IACF patients had fracture classification Sander type 1 (38.7%) and Sander type 3 (32.3%). Out of 62 patients, 38 patients (61.3%) were the displaced IACF who had been treated operatively; and the remaining 24 patients (38.7%) were the non-displaced IACF patients who had been treated non-operatively. These patients were used to compare the results with the operated group. 88.7% of patients were good compliance of no weight bearing at least 10 weeks and 35.5% of patients able to RTW or ADLs within 14 weeks. The mean Bohler angle for fractured foot (26.42°) was lower compared to normal foot (31.81°) whereas the mean Gissane’s angle was higher in fractured foot (137.42°) compared to normal foot (131.87°). The radiology result showed 59.7% anatomical, 27.4% near anatomical and 12.9% approximate/failure among all the IACF patients after treatment. The outcome of all the IACF patients in this study showed a good MFS with median of 83.50 and IQR of 17.00. The SF-36v2 PCS score among patients was below average with median 45.30 (IQR 15.13) whereas the SF-36v2 MCS score was average with median 56.60 and IQR of 9.20.

**Table I:** Characteristics of all the IACF patients (n=62)

Variables		Frequency (%)	Median (IQR)
Age			40.00 (18.00)
Gender			
	Male	52 (83.9)	
	Female	10 (16.1)	
Ethnic			
	Malay	26 (41.9)	
	Chinese	17 (27.4)	
	Indian	4 (6.5)	
	Bumiputera	15 (24.2)	
Occupation			
	Heavy labourer worker	8 (12.9)	
	Non-heavy labourer worker	54 (87.1)	
Mechanism of injury			
	Fell down from heights	40 (64.5)	
	MVA	40 (64.5)	
	Low-energy injury	7 (11.3)	
Fractured foot			
	Right	45 (72.6)	
	Left	17 (27.4)	
Classification			
	Sanders type I	24 (38.7)	
	Sanders type II	14 (22.6)	
	Sanders type III	20 (32.3)	
	Sanders type IV	4 (6.5)	
Treatment			
	Non-operative	24 (38.7)	
	Operative	38 (61.3)	
Days before operation/POP (day)			6.00 (3.00)
Days of hospitalization (day)			10.50 (7.00)
Ankle immobilization			
	< 2 weeks	36 (58.1)	
	> 2 weeks	26 (41.9)	
Weeks begin full weight bearing (week)			14.00 (2.00)
Compliance no weight bearing			
	poor, WB earlier than 10 weeks	7 (11.3)	
	good, NWB >10weeks	55 (88.7)	
Weeks before able RTW/ADLs			
	< 14 weeks	22 (35.5)	
	> 14 weeks	40 (64.5)	
Post-treatment duration assessment (month)			18.50 (16.00)
Normal foot Bohler’s angle (degree)			*31.81 (3.43)
Fracture foot Bohler’s angle (degree)			*26.42 (4.26)
Normal foot Gissane’s angle (degree)			*131.87 (4.61)
Fracture foot Gissane’s angle (degree)			*137.42 (6.23)
Radiology result			
	Anatomical	37 (59.7)	
	Near anatomical	17 (27.4)	
	Approximate/failure	8 (12.9)	
Maryland Foot Score			83.50 (17.00)
	Excellent (90-100)	22 (35.5)	
	Good (75-89)	25 (40.3)	
	Fair (50-74)	12 (19.4)	
	Failure (<50)	3 (4.8)	
SF-36v2 PCS score			45.30 (15.13)
	Below average (<49.99)	40 (64.5)	
	Average (≥50)	22 (35.5)	
SF-36v2 MCS score			56.60 (9.20)
	Below average (<49.99)	15 (24.2)	
	Average (≥50)	47 (75.8)	

* mean (SD)

Comparison between those non-operative and operative treatment patients, only 11 (17.7%) of 26 patients (40.2%) had anatomical reduction and fixation of fractures with no articular incongruence (Table II). There was no significant mean difference of MFS found between non-operative and operative treatment groups. Operative treatment group had no significant higher mean MFS compared to the non-operative treatment group (p=0.172). For both the SF-36v2 PCS and MCS scores, there were no significant mean difference between non-operative and operative treatment groups; the p-value respectively was 0.523 and 0.410. The operative treatment group had no significant higher mean SF-36v2 PCS and MCS score compared to non-operative treatment group. In addition, the difference of weeks before the patients were able to RTW or ADLs was compared between the two treatment groups. There was no significant mean difference of weeks before able to RTW or ADLs between non-operative treatment and operative treatment groups. Operative treatment patients had no significant shorter weeks before able to RTW or ADLs (p=0.264).

**Table II:** Distribution of Results and Outcomes between Non-operative and Operative treatment groups

Variables	Treatment			
	Non-operative Frequency (%)	Operative Frequency (%)	Total (%)	
Radiological Results				
Anatomical	11 (17.7)	26 (40.2)	37 (59.7)	
Near anatomical	10 (16.1)	7 (11.3)	17 (27.4)	
Approximate/failure	3 (4.9)	5 (8.0)	8 (12.9)	
	Mean (SD)	Mean (SD)	t-statistics (df)	p-value
*Maryland Foot Score	76.54 (14.59)	81.97 (15.39)	-1.38 (60)	0.172
*SF-36v2 PCS score	42.54 (8.9)	44.26 (11.06)	-0.64 (60)	0.523
*SF-36v2 MCS score	52.77 (9.56)	54.65 (8.16)	-0.83 (60)	0.410
*Weeks before able RTW or ADLs	16.63 (4.21)	15.53 (3.42)	1.127 (60)	0.264

* One Sample T-test

There were significant correlations between fractured foot Bohler angle and outcomes. Fractured foot Bohler angle had a significant positive correlation with MFS (Pearson correlation=0.61, p<0.001). Fractured foot Bohler angle had a significant positive correlation with SF-36v2 PCS score (Pearson correlation=0.57, p<0.001). Fractured foot Bohler angle had a significant positive correlation with SF-36v2 MCS score (Pearson correlation=0.48, p<0.001) (Table III).

**Table III:** Pearson’s correlation between outcomes and fractured foot Bohler’s angle

Outcomes	Fractured foot Bohler’s angle	
	Pearson’s correlation	p-value
Maryland Foot Score	0.61	<0.001
SF-36v2 PCS score	0.57	<0.001
SF-36v2 MCS score	0.48	<0.001

The associate factors of outcomes for IACF patients were performed by using simple linear regression was showed in Table IV. There were significant association found between occupation, compliance with non-weight bearing, month of post-treatment duration assessment, fractured foot Bohler angle, normal foot Gissane angle, fractured foot Gissane angle, radiology result and MFS. Non heavy labourer worker showed significant higher MFS. Good compliance with no weight bearing more than 10 weeks had significant higher MFS. Increase of one month post-treatment duration assessment had increase 0.46 of MFS. Increase of one degree of fractured foot Bohler angle had increase 2.18 of MFS. Increase of one degree of fractured foot Gissane angle had reduces 1.63 of MFS. Anatomical radiology result had significant higher MFS.

**Table IV:** Associate factors of MFS for IACF patients using simple linear regression

Variables	Regression coefficient, b (95% CI)	t stat (df)	p-value
Age	0.12 (-0.18, 0.41)	0.80	0.427
Gender	-0.44 (-11.03, 10.14)	-0.08	0.934
Ethnic	1.17 (-2.07, 4.41)	0.72	0.473
Occupation	20.52 (10.18, 30.85)	3.97	<0.001
Mechanism of injury	-4.77 (-10.28, 0.75)	-1.73	0.089
Foot	-4.04 (-12.70, 4.63)	-0.93	0.355
Classification	-1.62 (-5.56, 2.33)	-0.82	0.416
Treatment	5.43 (-2.44, 13.30)	1.38	0.172
Days before operation/POP	-0.18 (-1.78, 1.42)	-0.22	0.827
Days of hospitalization	-0.12 (-0.87, 0.63)	-0.32	0.754
Ankle immobilization	-3.55 (-11.39, 4.28)	-0.91	0.368
Weeks begin full weight bearing	1.09 (-0.55, 2.73)	1.33	0.190
Compliance no weight bearing	18.70 (7.38, 30.01)	3.31	0.002
Weeks before able RTW/ADLs	-0.81 (-1.84, 0.22)	-1.58	0.119
Post-treatment duration assessment (months)	0.46 (0.15, 0.78)	2.98	0.004
Normal foot Bohler’s angle	-0.26 (-1.40, 0.88)	-0.45	0.652
Fractured foot Bohler’s angle	2.18 (1.45, 2.91)	5.98	<0.001
Normal foot Gissane’s angle	-1,33 (-2.11, -0.55)	-3.42	0.001
Fractured foot Gissane’s angle	-1.63 (-2.10, -1.16)	-6.95	<0.001
Radiology result	-13.51 (-17.72, -9.30)	-6.42	<0.001

Based on simple linear regression analysis with p-value less than 0.25 and variables considered for biological plausibility, 11 variables were included. The variables for selection were occupation, mechanism of injury, treatment, weeks begin full weight bearing, compliance with no weight bearing, weeks before able RTW or ADLs, month of post-treatment duration assessment, fracture foot Bohler angle, normal foot Gissane angle, fractured foot Gissane angle and radiology result.

After the forward, backward and stepwise likelihood ratio (LR) selection methods were applied for variable selection, 5 variables were automatically selected in multiple linear regression analysis. Based on principles of best fit, Parsimonious principle, biological plausibility and condition of statistical significant (p<0.05), backward model were chosen in this study and were found to give the main effect to the MFS outcomes.

Multiple linear regression analysis was used to identify few factors which affecting the general outcome of IACF. Detrimental host factors which can affect outcomes in this study had been eliminated and there was no bias in the surgeon factors because only one qualified Ankle and Foot surgeon had performed this surgery. There were 5 main factors found to be affecting the patient’s outcome

There was a significant positive linear relationship found between compliance of no weight bearing and the MFS (p=0.002). The patients who had well compliance of not weight bearing for more than 10 weeks had 20 times higher MFS outcome compared to those started early weight bearing before 10 weeks (95%CI 7.58, 32.39).

There was a significant negative linear relationship found between weeks before they were able to RTW and ADLs and the MFS (p=0.003). An increase of 1 week before they were able to RTW or ADL had reduced MFS by 1.51 (95% CI -2.49, -0.52). In other words, patient with higher MFS will be able to RTW and achieve ADL earlier.

There was a significant positive linear relationship found between post-treatment duration assessment and the MFS (p=0.003). Increase of 1 month post-treatment duration assessment had increase MFS by 0.31 (95% CI 0.11, 0.51). The patient’s symptoms and MFS outcomes will slowly improved (0.31/month) with time.

There was a significant positive linear relationship found between fractured foot Bohler angle and the MFS (p=0.005). Increase of 1 degree of fractured foot Bohler angle had increase MFS by 0.94 (95% CI 0.30, 1.59). The Bohler angle reflects the relationship of posterior facet relative to the tuberosity of the calcaneum. The flattening (decrease) of the fractured foot Bohler angle means that the calcaneal inclination was lost. By increase one degree of the fractured foot Bohler angle towards the normal range during surgery, can improve the MFS outcome by 0.94 marks.

The Gissane angle is rarely described by any authors and no study been done to correlate it with the clinical outcomes. In this study, surprisingly, there was a significant negative linear relationship between fractured foot Gissane angle and the MFS (p=0.007). Increase of one degree of fractured foot Gissane angle had reduce MFS by 0.71 (95% CI -1.22, -0.20). In other words, the broader (increase) the obtuse angle of Gissane, the lower the MFS score.

## Discussion

The population chosen for this study was from Sabah. The study subjects consist of few ethnics such as Malay, Chinese, Indian and other ethnic Bumiputera, which provides a closer reflection of Malaysian’s actual ethnic population. From this study, it show that the IACF can occur in a wide range of age distribution in a population and ascertained that the male was predominantly (83.9%) sustained this type of injury. About 41.9% of patients were Malays, followed by the Chinese (27.4%) and other ethnic Bumiputera (24.2%). Surprisingly, although 65% of these injuries were caused by falling from height, 87% of patients were non-heavy labourers. In addition, the right foot was most commonly injured (72.6%). The median follow up period was 18.5 months (range from 6 to 48 months).

In this study, all the patients with IACF (operative and nonoperatively treated) will be grouped according to the accuracy of anatomical features when compared with contra-lateral non-fractured foot radiographs during their latest follow up assessment. Besides considering the Bohler angle, the congruity of the subtalar joint was assessed. In the non-operative treatment group, 21 (87.5%) out of 24 patients had anatomical or near anatomical radiological results. Although this group of patients had an initially non-displaced fracture and treated non-operatively, there was still risk of re-displacement if they weight bear early. Similarly, 33 (86.8%) out of 38 patients in the operative treatment group had a stable reduction and fixation, maintained the anatomical or near anatomical radiological results. This similar result showed that the anatomical restoration surgery; the compliance of the patients towards rehabilitation regimens, and their no weight-bearing until evidence of union had been adhered. These became important for the good outcome of treatment.

The operative treatment group of patients achieved higher mean MFS but found to be no significant clinical difference as compared with the non-operative treatment group. It was the same insignificant findings found in both the SF-36v2 PCS and MCS scores as well. Even though most of the patients (87.1%) had achieved anatomical or near-anatomical radiological results, both the functional and general outcomes were only slightly better in the operative treatment group^25,28^. These equivocal results proved that the displaced fractures need to be anatomically reduced and fixed to achieve comparable results as those with non-displaced fractures.

Louck and Buckley^41^ found that the Bohler angle is a prognostic factor in displaced IACF. In this study, the Bohler angle showed significant clinical correlations with the all outcomes, namely MFS and SF-36v2 PCS and MCS scores (Table III). Table IV, showed that by restoration of one degree of Bohler angle in the fractured foot, the MFS increases by 2.18 marks. Surgical intervention to restore the height and the Bohler angle of the calcaneum was to prevent the heel pad “blow-out”. Fracture with higher level of comminution or smaller Bohler angles had poorer outcomes regardless of the treatment method^10^. Paul *et al.*^42^ reported that the poorest outcome in calcaneal fractures was seen in patients who were treated operatively without restoration of Bohler angle.

In this series about the RTW or ADLs, most of the patients in both treatment groups were hospitalized for median of about 10 days and treatment intervention was given within a median of 6 days. About 58% of all patients’ ankle was immobilized less than 2 weeks and about 89% of all patients had good compliance to not weight bearing for at least 10 weeks before radiological evidence of union. One third of the patients were able to RTW or have good ADL before 14 weeks after their injury. By comparison among the two treatment groups, there was no clinically significant mean difference of weeks before they were able to RTW or attain good ADL (Table II). This insignificant finding was true because most of the IACF patients, either non-operative or operative, will be treated by almost the same protective way of non-weight bearing with the anticipated risk of displacement or implant failure. Ankle immobilization for the soft tissue healing; to avoid fracture displacement in the non-operative group; and to prevent wound dehiscence in the operative group, risks causing ankle stiffness and painful gait. This could prevent patients from attaining early RTW or good ADL^1^.

Although operative treatment is considered the standard of care for many of the IACF, but the different surgical approach or method can yield different outcome. There are so many variables to be studied in relation to patient characteristics, fracture patterns and techniques; and comparison between non-operative and operative treatment is very difficult. Few factors such as open fractures, associated injuries, smoking, diabetes, vascular status and complications surrounding the treatment can be associated with poor functional outcomes. A larger scale, randomized, multicenter, controlled study involving surgeons well versed in both operative and non-operative fracture care of the IACF may answer this controversy. However, dilemma of ethical legality in surgical randomized clinical trial and conflicts of worker’s compensation and satisfaction are challenges for any study. The surgeon factor, surgical approaches and osteosynthesis perspective in this present study had been subjectively standardized in the expense of fracture characteristic. Besides that, the SF-36v2, which was used to assess patient’s general health status, can be affected variably by patient’s age, ethnicity, marital status, level of education, occupation and employment as well as the patient’s monthly income^43^. The rehabilitation regime and patient’s compliance were also variably affecting the patient’s outcome.

There were many short comings from this study, further more it was a retrospective study of outcome. There was presence of selection bias where the patients elected for surgery started with a bad calcaneal fracture. The assumption of a non displaced fracture to be managed non operatively was itself a flaw in the sampling. The study would have been better if a proper selection based on proper imaging was performed.

## Conclusion

Operative treatment for the displaced IACF must be seriously weighed on a case-by-case basis because infection or severe wound complications develop after surgery are usually worse than if they had been treated in closed fashion. The indications for operative treatment should be based on fracture displacement, degree of intra-articular involvement, status of the soft tissue envelope, and confounding host factors. The displaced IACF need to be anatomically reduced and fixed to achieve a slightly better functional and general outcome as compared with the group of non-displaced IACF patients which had been treated non-operative. The findings in this study can allow a surgeon or the managing team to have a concrete evidence based reason in advising a displaced fracture patient to undergo anatomical surgical reduction and fixation.

By comparison among the two treatment groups, the operative treatment patients had no significant shorter weeks before they were able to RTW or ADLs. By increase one degree of the fractured foot Bohler angle towards the normal range, improves the patient’s outcome by 0.94 marks. However, increase of one degree of fractured foot Gissane angle had reduced the patient’s outcome by 0.71 marks. Patients who had complied not weight bearing for more than 10 weeks had 20 times better outcome compared to those started early weight bearing before 10 weeks. Patients will be able to RTW and ADLs earlier if they achieved better outcome scores. In addition, the patient’s symptoms and outcomes will slowly improve (at the rate of 0.31/month) with time.
